# Correspondence

**Published:** 1876-05

**Authors:** 


### Correspondence.

IRVING, N. Y., March 6, 1876.

*To Wm. H. Byford, M.D.:*

SIR—The case of Milton Brooks which I present you for publication is one which has awakened great interest among the medical men of Chautauqua county, and I doubt not will interest the readers of your Journal in the western part of our country. Perhaps Dr. Rogers will report it in an Eastern journal, but I think he has not obtained a full and perfect history of the case.

A very remarkable case of what has been called extreme costiveness by the physicians who have attended him at various times during a long period of sickness and suffering, has recently come under my observation. Believing it to be a case which will be of deep interest to the medical profession, I propose to give a brief history of it for publication in your Journal.

Milton Brooks, now deceased, had been the subject of a distressing complaint from the age of two years up to his death, which took place at the age of about 26 years. I have obtained the following statement from his parents. While an infant, nearly two years of age, he suffered from diarrhœa which became chronic and defied medical treatment for several months, but yielded at last, and was immediately followed by constipation, which condition of the bowels continued up to his death, but varied in their time of movement, the evacuations varying in time from two weeks, or about that, up to eight months and sixteen days. It was quite common for three, four and five months to intervene between evacuations.

During this great length of time this condition of his bowels continued without much mitigation, if any, from medical treatment, and that was often resorted to. He was treated by several physicians during his illness. Dr. Harrison, of this county, treated him mostly while in youth. Subsequently, Dr. Strong, of Westfield, Dr. Rogers, of Dunkirk, Dr. Bishop, of Silver Creek, and during the last three months of his life, I attended him. While he was under my care, I obtained from him a more minute description of his symptoms from the time he was old enough to understand and remember particulars about his sickness and suffering. I learned from him, as well as from his parents, that he suffered extremely every time he had evacuations of feces, often several hours, notwithstanding the evacuations were thin.

Generally, after his evacuations, pain gradually passed off and he would feel quite comfortable, eat quite hearty and do some light work, until he was again distended from the accumulation of feces in the colon. The accumulation being gradual, his body seemed to accommodate itself to the condition, but he was obliged to eat less as the distention increased, but at last the pressure on the stomach appeared to interfere with the functions of digestion, then his food would enter into fermentation, gas would generate rapidly, severe distress would commence, eructation would follow, and sometimes vomiting. The instincts of nature would be awakened and he would press and knead his bowels. In this way he would procure evacuations, and I concluded from his own account and my own observations during my attendance of him, that he never had a natural evacuation by the unaided efforts of nature, but always from gas, pressure and kneading of the bowels. The evacuations were enormous when the intervals without a passage were extended several months.

After the time that he went eight and a half months, he was weighed before the evacuation, and a few days after, and his weight was forty pounds less. This, to strangers to the case, would appear incredible, but we who know the character of the young man and that of the parents do not doubt the statement. Notwithstanding this abnormal condition through his whole life from infancy, his system was well nourished, and developed into an average-sized man. The only time he was comfortable was from the time of evacuations until the pressure from accumulation upon his stomach, his lungs and his heart took place; then he suffered continually until he had evacuations. After his growth was completed he became quite fleshy; quite a large accumulation of adipose had taken place about the intestines and under the skin. The great pressure from the accumulations produced considerable deformity of his body or disproportion, as the measurements at different times have shown.

He was never able to do any hard work. He could not run, or get around in a hurried manner like young men in general. He was always moderate in his movements. He was pale and exsanguine. His intellectual faculties were well developed, and I should judge he had a fair common school education.

This case being decidedly a remarkable one it attracted the attention of several medical men of this county who had the privilege of investigating it. He has several times been brought before the medical society of this county at the annual meeting, for examination. Dr. Rogers, of Dunkirk, in this county, reported the case to the members of the faculty at the annual meeting of the American Medical Association or a State Association, (I am not positive which), I think nearly four years since, as it then existed.

Dr. Strong, of Westfield, reported the case in the *Philadelphia Medical Journal* in October, 1874, as he found it at that time. His report is as follows:

"Milton Brooks, residing in Sheridan, Chautauqua Co., New York; age, 24; height, 5 feet, 10 inches; unloaded weight, 125 pounds; skin pale, and has a waxy look, tongue clean and pale; has been habitually constipated from childhood. The first medical history of him which I have obtained is by Dr. Geo. S. Harrison, of Sinclairville, who attended Brooks when two years old for costiveness. His habit then was to go about two weeks without fecal evacuations.

Several years later he attended him for diphtheria, and the time was then extended to six weeks. Dr. H. has occasionally seen him and watched the case up to this time, and says the disease has been gradually increasing. For the last six years he has been under my observation and has several times been before our medical association where careful measurements and examinations have been made. Most of the medical men of this county know more or less of the case. In May, 1872, I took the following measurements of him naked :

One girth above the nipple, 34½ inches ; one girth at umbilicus, 36 inches ; one girth 2 in. above umbilicus, 37 inches ; one girth 3 in. above umbilicus, 39 inches ; one girth 4 in. above umbilicus, 39½ inches ; one girth 6 in. above umbilicus, 39 inches ; one girth at lower end of sternum, 38 inches. Apex beat of heart one inch above nipple and one and a half inside.

March, 1874, I took the following notes :

Four weeks after he had a partial evacuation, and four months before he had a complete one. Girth at umbilicus, 37½ inches ; girth at 4 in. above umbilicus, 38½ inches ; girth at 6 in. above umbilicus, 38½ inches ; girth at epigastrium, 38 inches ; girth at nipple, 36½ inches.

Dr. Bishop, of Silver Creek, who is now B.'s adviser, wrote me, May 7, 1874 : His bowels have moved a little several times since you saw him at my house, March 14. Once or twice a year he has had an attack of vomiting attended with violent pain in the stomach and bowels, since I have been his medical adviser.

In 1872 he had such an attack which was controlled by hypodermic injections of morphine, and again in 1873 the same occurred and bowels were completely evacuated. The process of defecation lasts from two to four days, at which time he is sick and becomes much exhausted ; the dejections look like brown paper chewed.

There is free escape of gas per anum. The weight of fecal matter at one dejection was approximately obtained. He was accidentally weighed just before the movement and again as soon after as he could get to the scales. The difference was forty pounds. The longest interval between any fecal discharges occurred four years since, and was eight months and sixteen days. His abdomen when loaded is hard, the colon immensely distended and easily traceable.

He is a laboring man, does light work on a farm in a moderate way. He has been under the care of many physicians of all kinds, both intelligent and otherwise, and every imaginable treatment, followed by no permanent benefit.

P. S. Since writing the above report, Dr. Bishop writes me that he has pursued the course of treatment agreed upon by us last spring, and has given to B. the elixir of cinchona, iron and strychnine, with occasional small doses of calomel, the result of which has been evacuations at shorter intervals, so that his body was diminished somewhat. From this date given by Bishop, 1874, down to the present time (I do not mean the date of this report) but the time he first called me to treat him, there has been no material change in his symptoms."

The treatment agreed upon by Doctors Strong and Bishop only improved his condition for a few months. Mr. Brooks called on me some two months previous to his death. He first complained of pain in his back, in the region of the kidneys, of loss of appetite and occasional nausea. His tongue was covered with a yellow fur, and his urine was scanty and had a dark, dirty appearance, without much, if any, sediment. I gave a few doses of hyd. cum creta, with Dover's powders and nitre and buchu ; he appeared languid, and was inclined to keep the bed a part of the time, through the day. Some eight or ten days later I was called again ; found him in great pain in the bowels, and in the left side in the region of the heart ; his whole body appeared full and hard ; he occasionally vomited. I immediately gave morphine hypodermically, which gave prompt relief, but the anodyne treatment had to be continued for about two days, during which time his bowels were well evacuated. In a few days he was up and about as usual. In about two weeks he had another similar attack and was relieved by the same treatment, but during this time his tongue and urine gave evidence of deranged secretions ; in about ten days he began to suffer with severe pain in his head ; it came on in the morning and passed off at evening, and was attended with increased circulation and heat; his thirst was extreme ; his pulse ran from 110 to 120. I gave him the treatment that I judged his disease indicated. In about a week he recovered, a good appetite followed, and he gained strength quite rapidly, eating largely of hearty food, which he generally craved. About ten days subsequent to this attack, a severe pain in the stomach commenced, followed by great distention of his entire body, with copious evacuations. The pain soon reached his heart. I gave him an hypodermic injection of acetate morphia, followed by large doses of tincture of opium, which gave him considerable relief for about twelve hours, but the evacuations continued.

Pain returned, with greater severity about the heart and lungs, his respiration soon become labored, got no relief from anodynes, and in about forty-eight hours from this attack death came to his relief.

This attack was quite similar to those which he had while under my care every time he had evacuation from the bowels, only much severer, and he sank under the exhausting effect of the pain. He called these attacks diarrhœa. From the knowledge I obtained from my own observation and from Mr. Brooks, I came to the conclusion that he never had fecal evacuations except from accumulations of gas, generated in the stomach.

This brings us to the post-mortem examination which was had the day after his death, (January 22, 1876). Called Dr. Strong, of Westfield, Dr. Rogers, of Dunkirk, and Dr. Thompson, of Angola. The first part examined was the colon. That which first presented, was what appeared to be the ascending transverse and descending colon. It presented nearly a smooth surface, with but slight, if any, evidence of the longitudinal and circular bands that characterize it in its normal state. On examining it minutely, it was found to extend into the thorax at least four inches above its natural position, crowding the heart and lungs up before it to a transverse line one inch above the nipples ; its length was six feet and three inches ; its circumference was, in an undistended state, thirteen inches. It was not quite uniform in size, being the largest near its termination in the rectum. It diminished rapidly after passing over the prominence of the sacrum. About three inches below, the rectum was found contracted down to about one inch in diameter where the coats appeared thicker and more muscular. About six quarts of fecal matter were retained. The liver was 5½ inches thick and nearly double the natural size ; the spleen was considerably enlarged ; heart small ; stomach natural ; kidneys small ; lungs greatly compressed.

What appears the most remarkable in this case is the fact, that such an organic change should take place so suddenly in a young child as to oppose successfully the natural efforts of nature, and as he approached manhood the resistance increased until most of the internal organs were displaced or otherwise interfered with, and his entire body or thorax and abdomen permanently enlarged.

This case shows another fact that is remarkable. This condition was tolerated twenty-four years, was attended with great suffering, got no benefit from treatment, and still his system was fully developed. Was this hypertrophy ? was it paralysis ? was it the result of inflammation, and could he have been relieved had the case been understood ?

P. S. On looking over what I have written, I discovered I had overlooked an important part of the description of the post-mortem examination. Drs. Rogers and Strong did the work, while I took notes of the examination. When they had examined the colon and the liver, spleen, stomach and the situation of the heart and lungs, they appeared to be satisfied that they had discovered enough to account for the symptoms in the case. I suggested to them that I believed the cause of the obstruction was in the rectum which had not been disturbed, but the colon they had removed, which at the time contained 1½ gallons of thin feces. They did not heed my suggestion and said to the family that the examination was satisfactory, they had found all that was desirable. The second day after the body was interred, Dr. Rogers obtained leave of the parents to disinter the corpse and make a further examination. The rectum was then removed, which on examination was believed to unfold the mystery. It was apparently contracted when compared with the colon, but this was only apparent when its organic change showed that its muscular coat was greatly thickened. I should think its thickness was at least three-sixteenths of an inch, and in fact the whole rectum appeared thickened or hypertrophied and unyielding. A ball of hard fecal matter was found in the rectum about one inch in diameter. I have learned from the patient and his parents that his evacuations were always thin and without hardened feces preceding it—unlike all other cases.

---

CINCINNATI, March 13, 1876.

MY DEAR DOCTOR:

It seems proper that I should communicate to you some facts respecting the case of single cyst, about which I wrote you some time ago. I have just seen the patient, whose appearance and feelings are those of vigorous health. I was curious to know if there had been any new deposit of cystic fluid. Its presence is not very decided, but I am satisfied that refilling has commenced. In addition, I have detected an elongated and hardened tumor in the left ovarian region—a complication not before apparent.

It is now more than ten months since the last tapping, during which time the patient has not had a day of ill health, nor is there at this time any inconvenience from the presence of tumor or cyst.

The solidity of a part of the morbid growth has changed the aspect of the disease so far as relates to the cure. The long intervals between the emptying of the cyst by tapping, and the general good health of the patient, justified the belief that a formidable operation might not become necessary. The future is somewhat darkened, and in view of this fact, the practical question presenting itself is, what plan of treatment is best calculated to secure comfort and safety ?

On one point my friends of the knife differ in opinion. One advocates an operation before the morbid growth has attained any considerable size, and before the general health has become impaired. Another urges postponement until the entire system becomes more or less involved in the mischief.

The former view has the stamp of plausibility, and yet, cases of the most encouraging character, in the hands of skillful operators, have terminated fatally. Others, again, in which there was every evidence of failing health, the operation requiring extensive tearing and cutting, have survived the shock. We are then without sufficient data upon which to form a positive prognosis.

Is it not the wiser plan, in cases of single or even double cyst, to evacuate the fluid by tapping rather than run the certain hazard of ovariotomy ? The patient to whom I have more special reference is in good general health, and will probably remain so until the cyst enlarges, so as to produce mechanical inconvenience. An evacuation of the fluid will be followed by a return of good feeling. Thus may the life and usefulness of the patient be prolonged many years. Such are my inducements for opposing the formidable operation of removal of tumor and cyst.

Truly, M. B. WRIGHT.

DR. W. H. BYFORD, Editor, etc.

